# Supplementary material for: Differences in Biofilm Formation by Methicillin-Resistant and Methicillin-Susceptible Staphylococcus aureus Strains
Source: Diseases. 2023 Nov 5;11(4):160. doi: 10.3390/diseases11040160 (PMC10660471; doi:10.3390/diseases11040160)
Supplement: Supplementary file 1 [file diseases-11-00160-s001.zip › diseases-2641659-SI.pdf]

Supplementary Table 1. Source of the *S. aureus* isolates, and the biofilm detection method (BDM) used in the analysis. CV, crystal violet staining. CR, Congo Red Method.

Ref.	Number and source of the *S. aureus* isolates	Medium	BDM
[7]	27 clinical isolates (pus, blood, urine, sputum, urethral swabs, pleural fluid, cerebral spinal fluid) from a Hospital in Nepal.	TSB	CV
[8]	75 clinical isolates from urinary tract infections from 4 hospitals in Theran, Iran.	TSB 1% gluc.	CV/CR
[9]	197 clinical isolates from children with infections, Theran, Iran.	TSB	CV/CR
[10]	163 samples from nasal swabs, blood, and wounds from the National Reference Center of the Israel Ministry of Health	TSB 0.25% gluc.	CV/CR
[11]	50 samples from pus/burns swab samples from an Intensive Care Unit Hospital, Hamadan, Iran.	TSB 1% gluc.	CV
[12]	135 pus/wound swabs from skin and soft tissue from a Care Center, Theran, Iran.	TSB 1% gluc.	CV/CR
[13]	130 clinical isolates from wound, nose, throat, catheter, blood, urine, sputum, bronchoalveolar washings from hospitals in Siedlce and Warsaw, Poland.	TSB 0.5%	CV
[14]	143 clinical samples from admission patients, from 2 hospitals in Babol, Iran.	TSB 1% gluc.	CV
[15]	209 clinical samples (trachea, blood, wound, sputum, soft tissue, bronchus) from different hospitals, Theran, Iran.	TSB 1% gluc.	CV
[16]	35 MSSA and 22 MRSA isolates from nasal swabs from patients and medical students in a Hospital from Amman, Jordan.	TSB 2% gluc.	CV/CR
[17]	302 MRSA/ 268 MSSA isolates from carriers or infections from patients hospitalized in a Hospital in Gdansk, Poland.	TSB 1% gluc.	CV/CR
[18]	50 MRSA and 50 MSSA from clinical isolates from Cairo Univ. Hospital, Egypt.	TSB 1% gluc.	CV
[19]	114 MRSA and 98 MSSA isolates from device-related infections from a Hospital in Dublin, Ireland.	BHI 1% gluc.	CV
[20]	32 isolates (sepsis) from an Intensive Care Unit in a Hospital in Dublin, Ireland.	BHI 1% gluc.	CV
[21]	100 isolates from blood, urine, wounds, and catheter infections) from a Hospital in Hamadan, Iran.	Not mentioned	CV
[22]	76 isolates from pus/wound swabs from a Hospital in Lalitpur, Nepal.	TSB 2% gluc.	CV
[23]	92 clinical isolates from 3 different hospitals in Thailand.	TSB 0.25% gluc.	CV
[24]	217 clinical isolates (pus, sputum, blood, body fluid, urine) from a Hospital in Kathmandu, Nepal.	BHI 1% glucose	CV
[25]	Nasal swabs from volunteer healthcare personnel and medical students from different hospitals in Kathmandu, Nepal.	Not mentioned	CV
[26]	300 isolates from catheter-associated infections, wound and soft tissue, and urinary tract infections from 3 hospitals in Hungary.	TSB	CV/CR
